# Dysbiosis of gut microbiota and decreased propionic acid associated with metabolic abnormality in Cushing’s syndrome

**DOI:** 10.3389/fendo.2022.1095438

**Published:** 2023-01-23

**Authors:** Qin Zhang, Wen-mu Hu, Yu-ling Deng, Jin-jing Wan, Yu-jun Wang, Ping Jin

**Affiliations:** ^1^ Department of Endocrinology, The Third Xiangya Hospital of Central South University, Changsha, Hunan Province, China; ^2^ Health Management Center, The Third Xiangya Hospital, Central South University, Changsha, Hunan Province, China

**Keywords:** Cushing’s syndrome, *gut microbiota dysbiosis*, SCFAs (short chain fatty acids), propionic acid, gut, metabolic syndrome

## Abstract

**Objective:**

Chronic hypercortisolism leads to a phenotype resembling metabolic syndrome. We aimed to investigate the association between gut microbiota and metabolic abnormalities in endogenous hypercortisolism (Cushing’s syndrome).

**Methods:**

A total of 23 patients with Cushing’s syndrome (18 female and 5 men, aged 47.24 ± 12.99 years) and 30 age-, sex-and BMI-matched healthy controls (18 female and 12 men, aged 45.03 ± 6.69 years) were consecutively recruited. Differences in gut microbiota and plasma short-chain fatty acid (SCFAs) concentrations between the Cushing’s syndrome patients and controls were analyzed by 16S rRNA sequencing and gas chromatography-mass spectrometry (GC-MS).

**Results:**

Compared to the controls, the Simpson and Pielou indices of α diversity were dramatically decreased in Cushing’s syndrome (*P* < 0.05). The gut microbiota community structure differed significantly between Cushing’s syndrome patients and controls. Compared to controls, the bacterial communities of the Cushing’s syndrome patients were enriched in *Proteobacteria* and *Escherichia-Shigella*, and depleted in *Firmicutes*, including *Agathobacter*, *Blautia*, *Anaerostipes*, *Eubacterium_eligens_group*, and *Lachnospira*. Spearman analysis demonstrated that HbA1c, SBP, DBP, and cortisol levels were significantly positively correlated with *Proteobacteria* and *Escherichia-Shigella*, whereas negatively correlated with *Agathobacter, Blautia, Anaerostipes, Eubacterium_hallii_group, and Lachnospira*, etc. Cushing’s syndrome patients also had a lower propionic acid concentration (0.151±0.054 *vs.* 0.205±0.032 µg/mL, *P*=0.039) than controls. Furthermore, the level of propionic acid was negatively correlated with systolic pressure and cortisol levels (*P*<0.05).

**Conclusion:**

Gut microbiota dysbiosis and decreased propionic acid levels were observed in patients with Cushing’s, suggesting that the gut microbiota may be a potential therapeutic intervention target to improve hypercortisolism-related metabolic abnormalities.

## Introduction

Glucocorticoids are widely used as anti-inflammatory and immunosuppressive drugs in many immune-mediated diseases and for preventing rejection after organ transplantation. In addition, the administration of corticosteroids improves the survival of patients with severe COVID-19 (1); thus, glucocorticoids have greatly increased during the COVID-19 pandemic. However, chronic hypercortisolism leads to a phenotype that resembles metabolic syndrome, such as abdominal obesity, hypertension, and impaired glucose tolerance, which is a serious threat to patient health and limits the wide application of glucocorticoids (2).

Chronic hypercortisolism involves increased use of endogenous glucocorticoids (Cushing’s syndrome) and exogenous long-term/high-dose glucocorticoid use (iatrogenic Cushing’s syndrome). Excess endogenous glucocorticoids result in a severe condition known as Cushing’s syndrome (CS). Although surgery is the most efficient way to improve and cure hypercortisolism-related comorbidities, treating patients with unresectable neoplastic lesions or hidden ACTH-secreting lesions is limited (3). Meanwhile, persistent diabetes and hypertension after surgery in some patients will increase the mortality rate (4). However, therapeutics to ameliorate hypercortisolism-related metabolic dysfunction are still in preclinical development or clinical phase I and II trials (5). Adverse reactions, such as increased levels of adrenocorticotropic hormone or a potential nervous system, have also been reported (5). Therefore, exploring potential intervention targets may provide new strategies for preventing and treating hypercortisolism-related metabolic abnormalities.

Gut microbiota plays a central role in regulating host metabolism and has been reported to be associated with the development of various metabolic diseases such as obesity, diabetes, and cardiovascular diseases (6). To date, reports on gut microbiota and hypercortisolism-related metabolic abnormalities are rare. A recent study revealed that long-term prednisone treatment affects gut microbiota composition and fecal metabolites in mouse models. For instance, in rats treated with 6-week prednisone, the relative abundances of the genera *Eisenbergiella* and *Alistipes* decreased, whereas that of Anaerobacterium increased significantly (7). Tourret et al. found decreased Bacteroidetes and increased Firmicutes levels in mouse feces after 14 days of prednisone treatment (8). Dongxu et al. recruited 28 patients with acute transverse myelitis who received standardized glucocorticoid therapy and found a dramatic decrease in gut microbial diversity and enriched of Firmicutes and depletion of Bacteroidetes in GC-induced obese individuals (9). However, there have been no reports on gut microbiota and endogenous glucocorticoid excess. Herein, we explored the gut microbiota characteristics in patients with endogenous glucocorticoid excess (Cushing’s syndrome) to identify potential therapeutic intervention targets for hypercortisolism-related metabolic abnormalities.

## Materials and methods

### Ethics statement

This study was approved by the Institutional Ethics Committee of the Third Xiangya Hospital of Central South University (No. 22148). Written informed consent was obtained from all participants, and all procedures were performed according to the Declaration of Helsinki.

### Participants

Between January 2021 and September 2022, 23 patients with Cushing’s syndrome (18 women and 5 men, aged 47.24 ± 12.99 years) were consecutively recruited from the Third Xiangya Hospital of Central South University. Cushing’s syndrome was diagnosed according to the Pituitary Society 2021 guideline (10). The inclusion criteria were: 1) typical symptoms of Cushing’s syndrome, such as hyperemia, central obesity, and full moon face; 2) increased cortisol secretion, abnormal circadian rhythm, and twice 24­h urinary free cortisol (UFC) >45 µg/24 h; 3) impaired glucocorticoid feedback with overnight 1 mg dexamethasone suppression test (DST) and serum cortisol concentration ≥18 µg/dL (50 nmol/L); and 4) no exogenous glucocorticoids (oral, injections, inhalers, topical). Of the 23 patients, 18 had adrenocortical adenoma, 2 had Cushing’s disease, and 3 had adrenocorticotropin-independent (ACTH)-independent macronodular hyperplasia. Thirty geographically, age, sex, and BMI-matched healthy controls (18 women and 12 men, 45.03 ± 6.69 years). All healthy controls had normal cortisol and ACTH circadian rhythms and no history of diabetes, hyperlipidemia, or other endocrine diseases. All participants who fulfilled the following exclusion criteria were excluded:1) other organic or infectious diseases, such as immune system diseases, gastrointestinal disease, mental and psychological diseases, tumors, etc.; 2) taking steroids, anti-diabetic drugs, antibiotics, or probiotics three months before sample collection; 3) ectopic ACTH syndrome or adrenocortical carcinoma patients; 4) chronic alcoholics; and 5) any other conditions that may affect the gut microbiota, such as overeating or vegetarian for nearly a month.

### Biochemical assays

The clinical data of the Cushing’s syndrome patients were obtained from their medical records. Fasting blood samples were obtained after fasting for 12 h and were kept at -80°C. Fasting blood glucose (FBG), total cholesterol (TC), triglyceride (TG), high-density lipoprotein cholesterol (HDL-C), and low-density lipoprotein cholesterol (LDL-C) concentrations were quantified using an automatic biochemical analyzer (LST-008; Hitachi, Japan). Glycosylated Hemoglobin Type A1C (HbA1c) was detected using high-performance liquid chromatography (HA8180 automatic glycosylated hemoglobin analyzer). Cortisol and adrenocorticotropic hormone (ACTH) were measured using an electrochemiluminescence immunoassay (Roche Cobas 6000 automatic electrochemiluminescence analyzer), and 24 h UFC was detected using liquid chromatography-tandem mass spectrometry (Changsha Kingmed Center for Clinical Laboratory, China).

### 16S rRNA amplicon sequencing

Clean stools were collected from individuals before using any medication or DST, transported in ice bags, and stored at -80°C. Genomic DNA was extracted using the CTAB method, and the 16S V3-V4 region of the bacteria was amplified by PCR (Phusion^®^ High-Fidelity PCR Master Mix with GC Buffer, New England Biolabs). The NEBNext^®^ Ultra™ II DNA Library Prep Kit was used to construct the library, and NovaSeq6000 was used for sequencing. QIIME2 software was used to calculate Simpson and Pielou_e indices and Unifrac distance, and R software was used to draw PCoA and NMDS dimensionality reduction maps. Adonis and Anosim functions were used to analyze the significance of differences in community structure between groups. LEfSe software was used to complete the significantly different species analysis between groups. Random forest analysis was conducted to screen for biomarkers that play an important role in classification or grouping. PICRUSt2 was used to predict microbial metabolic function.

### Short-chain fatty acid analysis

Acetic acid, propionate acid and butyric acid (the charged modes were all positively charged) concentration of the plasma collected from different participants was performed using trace 1310 gas chromatograph (Thermo Fisher Scientific, USA) and ISQ LT (Thermo Fisher Scientific, USA). GC-MS instrument conditions: sample inlet temperature 250°C; Ion source temperature 230°C; Transmission line temperature 250°C, quadrupole rods temperature 150°C. Electron bombardment ionization (EI) source, full scan and SIM scan mode, electron energy 70eV. The peak area and retention time were extracted using MSD ChemStation software, and a standard curve was drawn to calculate the short-chain fatty acid concentration.

### Statistical analysis

Statistical analysis was performed using SPSS software 25.0. Data are presented as mean ± standard deviation (SD), median and interquartile range M (QL, QU), or proportion (%). The normality of distribution was assessed using the Shapiro-Wilk test. Statistical differences in normally distributed continuous variables were evaluated using an unpaired Student’s t-test. Statistical differences in non-normally distributed continuous variables were examined using the Mann-Whitney U test. Chi-square or Fisher’s exact tests were used to analyze differences in categorical variables. Correlation analysis was performed using Spearman analysis. All tests were two-tailed, and *P* < 0.05 was considered statistically significant.

## Results

### Clinical characteristics of Cushing’s syndrome patients and controls

The clinical characteristics of Cushing’s syndrome patients and controls are summarized in **Table 1**. No significant differences were observed in body mass index (BMI), age, or sex between the control and CS groups (**Table 1**). Compared to the controls, Cushing’s syndrome patients had higher HbA1c and TGs levels, lower HDL-C levels, and higher systolic and diastolic blood pressure. In addition, Cushing’s syndrome patients had a higher cortisol concentration and an abnormal circadian rhythm.

**Table 1:** Clinical characteristics of CS patients and controls.

variables	CS (n=23)	Controls (n=30)	RR
BMI (Kg/m^2^)	25.18 ± 3.00	24.17 ± 1.76	–
Age (year)	47.24 ± 12.99	45.03 ± 6.69	–
Sex (female/male)	18/5	18/12	–
SBP (mmHg)	152.64 ± 16.06*	113.30 ± 9.23	–
DBP (mmHg)	93.64 ± 16.47*	69.53 ± 6.44	–
FBG (mmol/L)	6.37 ± 3.38	5.31 ± 0.41	3.9-6.1
HbA1c (%)	6.60 ± 1.17*	5.36 ± 0.16	4.2-6.5
TG (mmol/L)	1.7 ± 1.28*	1.03 ± 0.37	<1.7
TC (mmol/L)	5.32 ± 1.80	4.90 ± 0.66	<6.22
HDL-C (mmol/L)	1.33 ± 0.24*	1.52 ± 0.33	1.29-1.55
LDL-C (mmol/L)	3.39 ± 1.08	3.00 ± 0.71	<3.4
Cortisol (8:00) (µg/dl)	18.12 ± 7.20**	11.62±1.90	5.72-19.42
Cortisol (16:00) (µg/dl)	17.30 ± 6.81***	7.43±1.25	2.02-13.1
Cortisol (0:00) (µg/dl)	16.43 ± 7.79***	4.00±0.89	2.02-13.1
24 h UFC (µg/24 h)	159.75 (80.95, 382.63)	–	3.5-45.0

CS, Cushing’s syndrome; BMI, body mass index; FBG, fasting blood glucose; TC, total cholesterol; TG, triglyceride; HDL-C, high-density lipoprotein cholesterol; LDL-C, low-density lipoprotein cholesterol; HbA1c, glycosylated hemoglobin; SBP, systolic blood pressure; DBP, diastolic blood pressure; 24 h UFC, 24­h urinary free cortisol. * P < 0.05; ** P<0.01; *** P<0.001 vs controls. RR, reference range.

### Diversity of gut microbiota in Cushing’s syndrome patients and healthy controls

**Figure 1:**
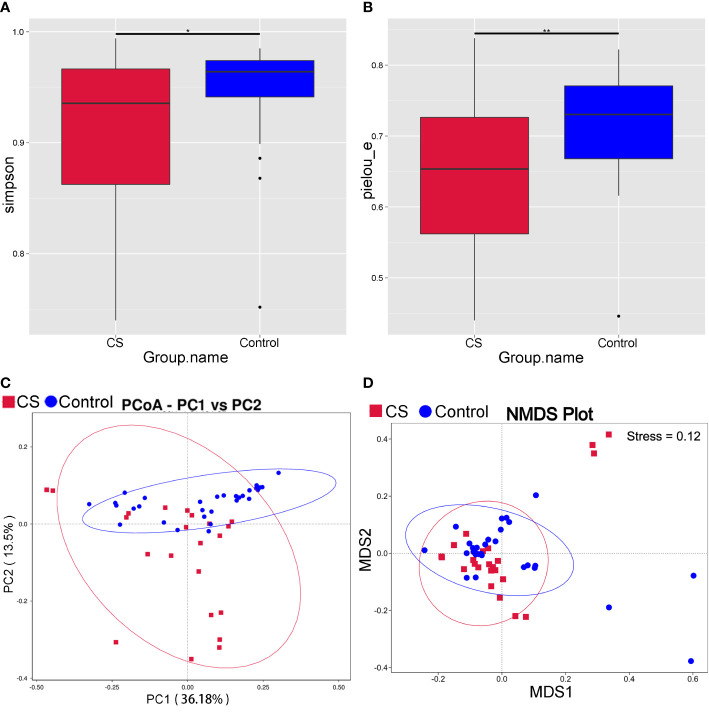
Analysis of intestinal microbiota diversity in Cushing’s syndrome patients and controls. **(A)**: Simpson index decreased in the Cushing’s syndrome group (*P* < 0.05); **(B)**: Pielou_e index decreased in the Cushing’s syndrome group (*P* < 0.01); **(C)**: PCoA analysis displayed that the Cushing’s syndrome group had a bacterial community composition distinct from that of the control group; **(D)**: NMDS analysis depicted that gut microbiota community structure of the Cushing’s syndrome group was different to the control group. CS, Cushing’s syndrome. *P<0.05; **P<0.01. PCoA,Principal coordinate analysis; NMDS, non-metric multidimensional Scaling.

Compared to controls, the Simpson and Pielou_e of α diversity index were significantly decreased in the Cushing’s syndrome group, which indicated that the species richness and evenness of the gut microbiota were decreased (**Figures 1A, B**, *P* < 0.05). The bacterial community composition was distinct from that of the control group according to principal coordinate analysis (PCoA) based on weighted UniFrac (**Figure 1C**). The gut microbiota community structure in the Cushing’s syndrome group was also different from that in the control group by non-metric multidimensional Scaling (NMDS) analysis (**Figure 1D**). There was a significant difference in the gut microbiota community structure of Cushing’s syndrome patients and controls by Adonis analysis (R2 = 0.081, *P*= 0.003) and Anosim analysis (R value= 0.151, *P*= 0.005).

### Differences in specific taxa between Cushing’s syndrome patients and healthy controls

**Figure 2:**
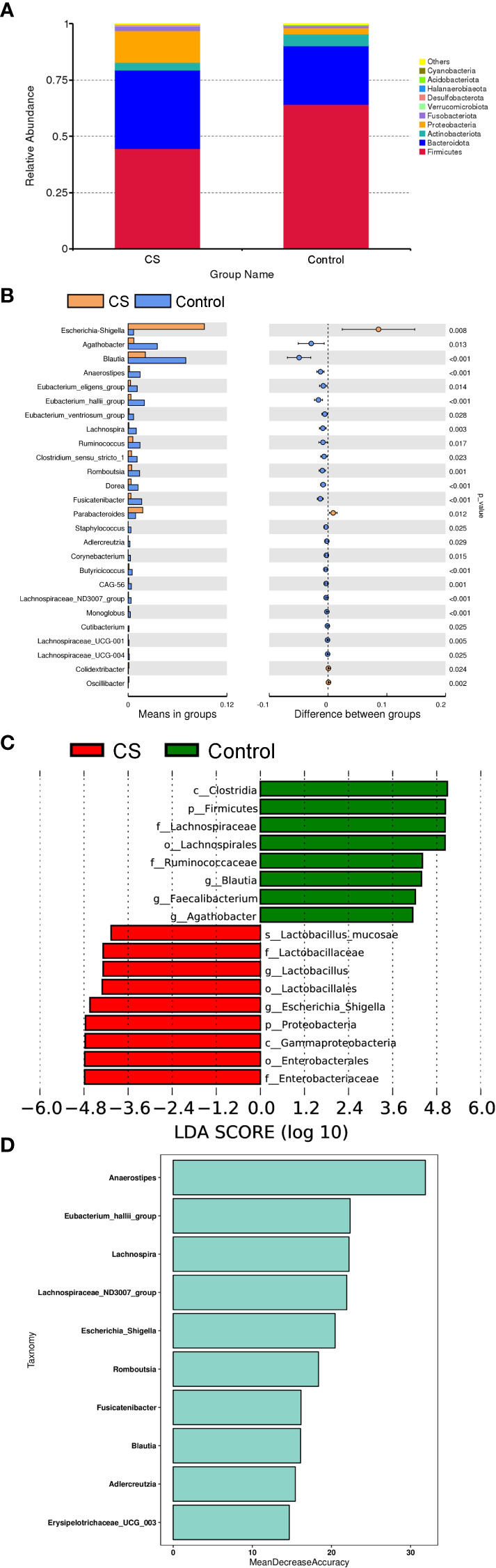
Difference in specific taxa between Cushing’s syndrome patients and controls. **(A)**: The relative abundance of gut microbiota at the phylum level showing that *Firmicutes* were significantly decreased in the CS group (*P*<0.001), while taxa in the *Proteobacteria* were increased (*P*<0.01). **(B)**: The relative abundance of gut microbiota at the genus level showing that the relative abundance of *Agathobacter*, *Blautia*, *Anaerostipes*, *Eubacterium_eligens_group*, and *Lachnospira* were decreased in the CS group (p <0.05), while the proportions of *Escherichia-Shigella* and *Parabacteroides* were enriched (p <0.05). **(C)**: LEfSe analysis demonstrated that *Escherichia-shigella* from *Proteobacteria* was a dominant genus in the gut microbiota of CS patients. **(D)**: Random forest analysis displayed the importance of specific taxa in groups. CS,Cushing’s syndrome; LDA,Linear Discriminant Analysis.

To further investigate differences in specific taxa between Cushing’s syndrome patients and controls, we analyzed the relative abundance of 16S rRNA reads at the phylum and genus levels. Compared to controls, the relative abundance of *Proteobacteria* was significantly increased, and the relative abundance of *Firmicutes* was significantly decreased at the phylum level in Cushing’s syndrome patients (**Figure 2A**, *P*<0.05). The *Firmicutes*/*Bacteroidetes* ratio was significantly decreased in the Cushing’s syndrome group compared to controls (*P*=0.025). Taxonomic analysis at the genus level demonstrated that the relative abundance of *Escherichia-Shigella* from *Proteobacteria* was significantly higher in Cushing’s syndrome patients *(P*<0.05), while the relative abundance of *Agathobacter*, *Blautia*, *Anaerostipes*, *Eubacterium_eligens_group*, *Eubacterium_hallii_group*, *Ruminococcus*, *Lachnospiraceae_ND3007* and *Lachnospira* from Firmicutes were significantly lower in Cushing’s syndrome patients (**Figure 2B**, *P*<0.05). As displayed in **Figure 2C**, *Escherichia-shigella* from *Proteobacteria* was the dominant genus in the gut microbiota of Cushing’s syndrome patients, while the dominant bacteria genera in the control group were *Blauia*, *Faecalibacterium*, and *Agathobacter* from Firmicutes. Random forest analysis revealed that *Anaerostipes*, *Lachnospira*, *Lachnospiraceae_ND3007_group*, and *Escherichia-Shigella* were the four most important biomarkers (**Figure 2D**).

### Correlation analysis of gut microbiota and clinical indicators

**Figure 3:**
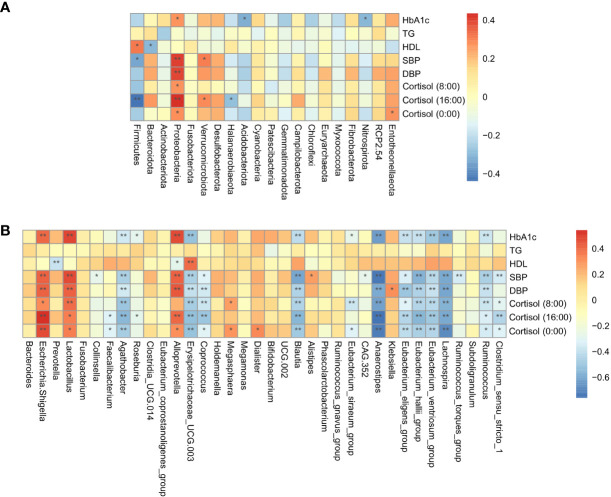
Correlation analysis of gut microbiota and clinical indicators. **(A)**: The correlation of specific taxa at the phylum level and clinical indicators between CS patients and healthy controls. **(B)**: The correlation of specific taxa at the genus level and clinical indicators between CS patients and healthy controls. TG, triglyceride; HDL-C, high-density lipoprotein cholesterol; HbA1c, glycosylated hemoglobin; SBP, systolic blood pressure; DBP, diastolic blood pressure *P < 0.05; **P<0.01.

Spearman correlation analysis was conducted to evaluate the relationship between gut microbial dysbiosis and clinical indicators in Cushing’s syndrome populations, showing that the HbA1c, cortisol level, SBP, and DBP were significantly positively correlated with *Proteobacteria*, *Escherichia-Shigella*, and significantly negatively correlated with the relative abundance of *Agathobacter*, *Blautia*, *Anaerostipes*, *Erysipelotrichaceae_UCG.003*, *Eubacterium_eligens_group*, *Eubacterium_hallii_group*, *Lachnospira*, *Ruminococcus*, etc. (**Figures 3A, B**).

### The predicted functional discrepancy in Cushing’s syndrome individuals and controls

**Figure 4:**
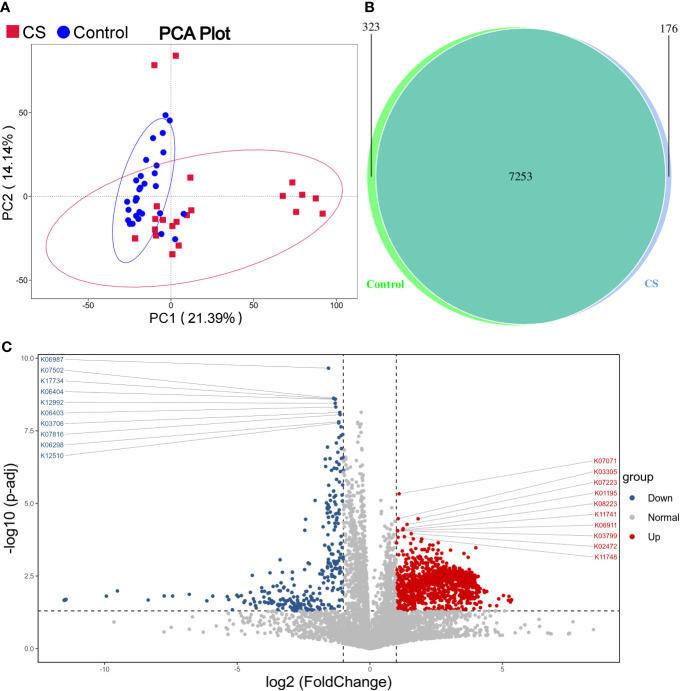
Function prediction analysis. **(A)**: PCA analysis revealed that the bacterial gene of the CS group might be different from the control group. **(B)**: Venn graph showing the number of genes in the two groups. **(C)**: Volcano map of differential genes. CS, Cushing's syndrome; PCA, Principal Component Analysis.

Functional prediction analysis using PICRUST2 was performed to predict the functional gene changes in the gut microbiota. PCA analysis revealed an obvious separation in the functional genes of the gut microbiota in the PC2 axis, which indicated that the bacterial genes might differ between groups (**Figure 4A**). The Venn graph depicted that the number of genes shared by the two groups was 7,253, with 176 genes unique to the Cushing’s syndrome group and 323 genes unique to the control group (**Figure 4B**). The volcano map demonstrated differential genes (**Figure 4C**).

### Serum short-chain fatty acids in Cushing’s syndrome patients and controls

Because the short-chain fatty acid-producing microbiota, such as *Blautia*, *Anaerostipes*, *Eubacterium_eligens_group*, *Eubacterium_hallii_group*, and *Lachnospira* were significantly decreased in Cushing’s syndrome patients compared to controls, the concentration of short-chain fatty acids was quantified in Cushing’s syndrome patients and controls by GC-MS. Compared to controls, propionic acid concentration was significantly decreased in the Cushing’s syndrome group (0.151±0.054 vs. 0.205±0.032 µg/mL, *P*=0.039) (**Figure S1**) and significantly negatively correlated with systolic pressure and cortisol levels (**Table 2**, *P*<0.05). There was no difference in butyric acid and acetic acid in those two groups (CS vs Control: butyric acid 0.041±0.049 vs 0.036±0.035 ug/ml, *P*=0.831; acetic acid 1.382±0.612 vs 1.517±0.512 ug/ml, *P*=0.664) (**Figure S1**).

**Table 2:** Spearman analysis of propionic acid and clinical indexes.

Items	r	95% confidence interval	*P*
TG	-0.1216	-0.5967 to 0.4166	0.6659
HbA1c	-0.3739	-0.7547 to 0.1955	0.1879
SBP	-0.6985	-0.8917 to -0.29	0.0038
DBP	-0.5041	-0.8078 to 0.011	0.0554
Cortisol (8:00)	-0.8036	-0.9343 to -0.4824	0.0005
Cortisol (16:00)	-0.6571	-0.8788 to -0.2024	0.0094
Cortisol (0:00)	-0.731	-0.9075 to -0.3349	0.0027

TG, triglyceride; HbA1c, glycosylated hemoglobin; SBP, systolic blood pressure; DBP, diastolic blood pressure.

## Discussion

In our study, the Cushing’s syndrome patients had decreased gut microbiota diversity and a significantly transformed bacterial community composition. Further analysis showed that the relative abundance of *Proteobacteria* significantly increased, while the relative abundance of *Firmicutes* decreased in Cushing’s syndrome patients. This differs from previous studies, which reported that the relative abundance of *Firmicutes* and *Actinomycetes* significantly increased, whereas *Bacteroidetes*, *Bifidobacterium*, and *Eubacterium* decreased in acute transverse myelitis (ATM) patients treated with prednisone (9). This difference may be related to the underlying disease (ATM) and the type of glucocorticoids used (prednisone), which may impact the specific gut microbiota taxa. The present study may accurately reflect the association between gut microbiota dysbiosis and hypercortisolism-related metabolic abnormalities because the endogenous glucocorticoid increase (Cushing’s syndrome) rules out the interference of other immune-mediated diseases using exogenous glucocorticoids.

The increased abundance of *Proteobacteria* in the gut is an important feature of gut microbial dysbiosis (11), which has been observed in obese and type 2 diabetes mellitus (T2DM) patients and high-fat-fed C57BL/6J obese mice (12, 13). Fei and Zhao (14) found that *Proteobacteria* and *Enterobacter cloacae B29* have a causative role in metabolic deterioration. Monocolonization of germ-free mice with *Enterobacter cloacae B29* isolated from a diabetic patient with extreme obesity can induce obesity and insulin resistance, which supports the relationship between metabolic disorders and the expansion of *Proteobacteria* (14).

The relative abundance of *Escherichia-Shigella* was significantly increased in Cushing’s syndrome patients, which has been reported to be associated with lipopolysaccharide (LPS) production (15–17). LPS can cause metabolic endotoxemia characterized by low levels of inflammation, insulin resistance, and increased cardiovascular risk (16). Furthermore, LPS can trigger the release of pro-inflammatory molecules, interfere with glucose metabolism, and prompt the development of atherosclerotic plaques and fatty liver disease (18). We also observed a significant positive correlation between *Proteobacteria*, *Escherichia-Shigella*, and the metabolic syndrome phenotype of Cushing’s syndrome patients, indicating an unstable gut microbial community characterized by an abundance of *Proteobacteria* and *Escherichia-Shigella* is a potential diagnostic signature of metabolic abnormalities in Cushing’s syndrome.

The relative abundances of members of *Firmicutes*, including *Agathobacter*, *Blautia*, *Anaerostipes*, *Eubacterium* spp. (eg. *Eubacterium_eligens_group* and *Eubacterium_hallii_group*) and *Lachnospira* were significantly decreased and negatively correlated with the metabolic syndrome phenotype of patiCushing’s syndrome patients. As previously reported, *Agathobacter* can produce butyrate and is involved in Rye-associated improvements in metabolic risk (19). A decreased abundance of *Agathobacter* was also observed in prediabetes (20). A recent study from Japan found that the *Blautia* genus, especially *B. wexlerae*, is a commensal bacterium that is inversely correlated with obesity and T2DM. Oral administration of *B. wexlerae* to mice can decrease high-fat diet-induced obesity and diabetes by producing succinate, lactate, and acetate (21). *Eubacterium hallii* is a butyrate- and propionate-producing bacterium (22, 23). Oral treatment with *Eubacterium hallii* improves insulin sensitivity and increases energy expenditure in obese and insulin-resistant db/db mice (24). *Anaerostipes* were significantly decreased in T2DM in African and European populations (25, 26) and can anaerobically convert inositol stereoisomers to propionic acid (27).

Since these genera have been reported to be related to SCFAs production, we further examined serum SCFAs levels and found a significant decrease in propionic acid levels in Cushing’s syndrome patients. This is consistent with previous studies showing a decreasing trend of propionate and butyrate content in the feces of obese patients induced by exogenous glucocorticoids (9). Propionic acid is one of the most common SCFAs produced by fermentation dietary fiber, which protects against diet-induced obesity and reduces food intake *via* satiety-inducing gut hormones (28). The anorexigenic effect of propionate is mediated through the GPR41/FFAR3 and GPR43/FFAR2 receptors in the intestine, which promote the secretion of GLP-1 and PYY peptides (28–31). Moreover, propionic acid stimulates the expression of the anorexigenic hormone leptin in human adipose tissue (32) and can rescue obesity caused by a high-fat diet by inhibiting Th17-mediated intestinal inflammation (33). Chambers et al. found that direct delivery of propionate in the proximal colon results in increased levels of PYY and GLP-1 and attenuated weight gain and adiposity in overweight individuals (34). Collectively, the decreased abundance of SCFA-producing bacteria and the level of propionic acid in patients with Cushing’s syndrome may be related to the development of hypercortisolism-related metabolic abnormalities.

Nonetheless, the present study had some limitations. This cross-sectional study failed to clarify the causal relationship between Cushing’s syndrome and gut microbiota. Further studies are required to verify the mechanism between Cushing’s syndrome and gut dysbiosis.

In conclusion, gut microbiota dysbiosis characterized by increased *Proteobacteria*, *Escherichia-Shigella*, and decreased SCFA-producing bacteria is a potential diagnostic signature of metabolic abnormalities in Cushing’s syndrome. Gut microbiota may be a potential therapeutic target to improve hypercortisolism-related metabolic abnormalities.

## Data availability statement

The original contributions presented in the study are publicly available. The 16S rRNA sequencing presented in the study are deposited in the SRA database, accession number PRJNA923180, and the GC-MS data is deposited in the MetaboLights database, MTBLS6875.

## Ethics statement

The studies involving human participants were reviewed and approved by The Institutional Ethics Committee of the Third Xiangya Hospital of Central South University (No. 22148). The patients/participants provided their written informed consent to participate in this study

## Author contributions

QZ performed experimental work. W-MH, Y-LD, J-JW, and Y-JW collected clinical data. PJ designed the study and drafted the manuscript. All authors contributed to the article and approved the submitted version.
