# Supplementary figures and images for: Dysbiosis of gut microbiota and decreased propionic acid associated with metabolic abnormality in Cushing’s syndrome
Source: Front Endocrinol (Lausanne). 2023 Jan 23;13:1095438. doi: 10.3389/fendo.2022.1095438 (PMC9901362; doi:10.3389/fendo.2022.1095438)

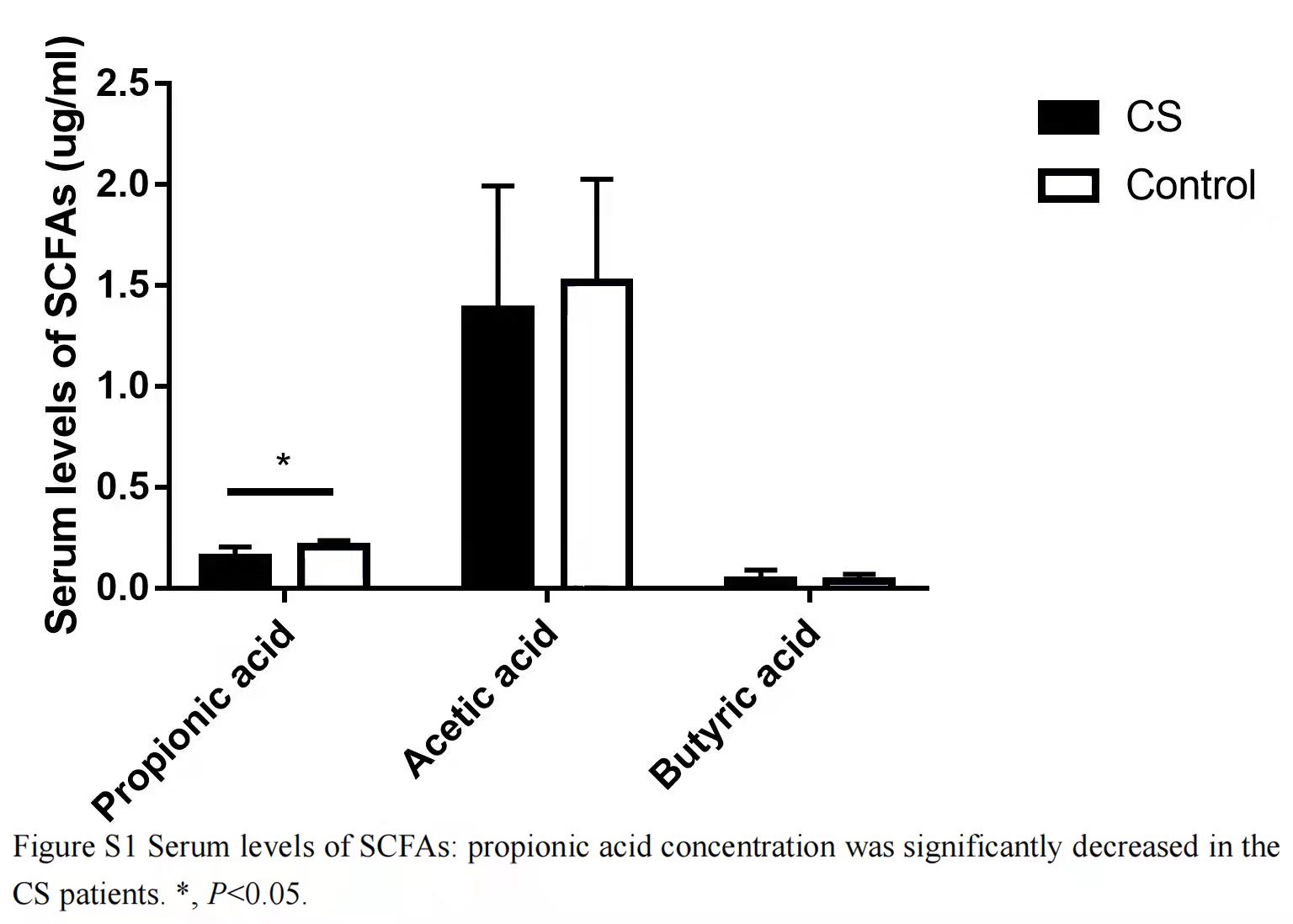

Supplement: Supplementary file 1 [file Image_1.jpeg]
